# Perchlorate‐tolerant bacterial strains isolated from the Mars‐analog Qaidam Basin soils exposed to Earth's near space

**DOI:** 10.1002/mlf2.12142

**Published:** 2024-09-16

**Authors:** Li Liu, Mengling Kang, Zhe Wang, Jianxun Shen, Yongxin Pan, Wei Lin

**Affiliations:** ^1^ Key Laboratory of Earth and Planetary Physics, Institute of Geology and Geophysics Chinese Academy of Sciences Beijing China; ^2^ College of Earth and Planetary Sciences University of Chinese Academy of Sciences Beijing China

## Abstract

Earth's lower near space of 20–40 km above sea level with polyextreme conditions serves as a unique Mars analog for astrobiological research to investigate the limits of life on Earth and planetary protection considerations for Mars exploration. In this study, we exposed Mars‐like desert regolith to near space at a float altitude of ~35 km and isolated four bacterial strains after exposure. In addition to stress tolerance to extreme environmental stressors, these strains represent a remarkable tolerance to perchlorate that is widespread in present‐day Martian soils. These extremophilic bacterial strains screened through near‐space exposure could serve as promising candidates for future astrobiological research in space stations or in laboratory‐based planetary simulation environments.

Earth's near space, defined as the region of 20–100 km above sea level (ASL), has a combination of strong ultraviolet (UV) and cosmic radiation, low temperature, extreme desiccation, hypoxia, and hypobaric conditions^1^. Lower near space (~20–40 km ASL) has been regarded as an ideal environmental analog to the present surface conditions of Mars^1^, ^2^. Astrobiological studies conducted in near space not only promote a greater understanding of life's strategies under polyextreme environmental conditions on Earth and beyond but also provide important implications for planetary protection considerations for Mars exploration^3^. Given that spacecraft landing on Mars may carry trace terrestrial organisms and interfere with the detection of life signals, extremophiles with potential tolerance to Martian shallow subsurface conditions should be of particular interest in terms of planetary protection policy and guidelines. To date, a group of pure cultures of bacteria, archaea, and fungi, either freeze‐dried or in culture media, has been exposed to near‐space Mars‐like environments to evaluate their survivability and adaptive strategies^4^, ^5^. However, to the best of our knowledge, few studies have investigated the survival of microbes colonized in natural samples (e.g., desert soils) after exposure to all stressors in Earth's near space, which could better simulate the true shallow subsurface conditions of present‐day Mars.

The Qaidam Basin in the northern Tibetan Plateau, China, is one of the driest (average annual precipitation: <45 mm) and highest‐altitude (average elevation: ~2800 m) deserts on Earth with a low annual temperature (average: 1–5°C) and widespread Mars‐like landforms and evaporites, and it has been considered as a representative terrestrial analog of the Mars^6^, ^7^. Microorganisms inhabiting the Qaidam Basin soils have been shown to be resistant to extreme terrestrial environments^8^. Halotolerant microbes have been discovered in the Dalangtan Playa of the Qaidam Basin, where perchlorates (ClO_4_^−^) are present in surface and subsurface soils^9^. As an easily soluble reactive oxidant, perchlorate can cause oxidative stress, osmotic stress, and DNA damage to most microbial life^10^, and it has been considered to be widespread on Mars^11^, ^12^. To date, many perchlorate‐tolerant microbes have been reported in terrestrial Mars analogs, such as the Atacama Desert hypersaline regions^13^ and the Antarctic Desert perchlorate‐rich brines^14^. These findings provide implications for potential halophilic life on Mars where perchlorate deposits and brines are available^15^. Thus, soil microbes in the Qaidam Basin are promising candidates for assessing microbial tolerance to polyextreme conditions and screening for pure cultures capable of withstanding Mars‐like environments.

In this study, in order to investigate whether soil‐dwelling microorganisms could survive in near‐space polyextreme conditions, soil samples from the Qaidam Basin were collected and exposed to near space (~35 km ASL) using a high‐altitude scientific balloon (Figure 1A). The BIOlogical Samples Exposure Payload (BIOSEP) with sample containers (Figure S1) was mounted on the Chinese Academy of Sciences Balloon‐Borne Astrobiology Platform (CAS‐BAP)^1^. The total float time of BIOSEP at 35 km was 4 h 11 min and the total exposure time of samples was 4 h (Figure 1B). The estimated UV radiation dose, cosmic radiation dose, atmospheric pressure, and relative humidity (RH) at the ~35 km altitude were ~100 W/m^2^, ~0.1 mGy/day, <1 kPa, and <1%, respectively, according to previous studies (Table S1)^2^.

**Figure 1:**
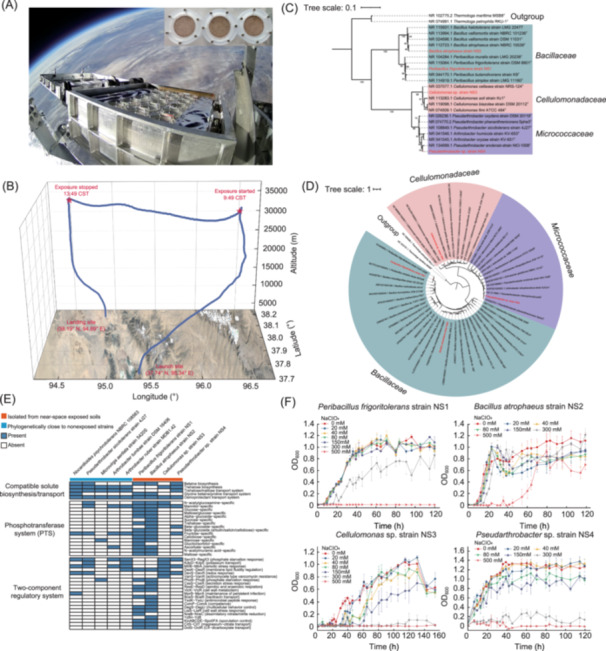
Near‐space exposure experiments and four perchlorate‐tolerant bacterial strains isolated from exposed desert soils. (A) Images of the BIOlogical Samples Exposure Payload (BIOSEP) during the exposure experiment at a float altitude of ~35 km and the sample containers mounted on the exposure payload. Soil samples were collected in the Qaidam Basin the day before the flight mission. (B) Travel map of the HH‐21‐5 flight mission. (C) Maximum‐likelihood phylogenetic tree based on 16S rRNA gene sequences. The sequences were aligned using MEGA (v.11) and the tree was constructed using IQ‐TREE under the TN + F + R3 substitution model. The number of bootstrap replications is 1000. (D) Phylogenomic tree constructed by PhyloPhlAn 3.0. The phylogenetic analysis was performed using 400 optimized universal marker genes. (E) Functional comparison of Kyoto Encyclopedia of Genes and Genomes modules involved in compatible solute biosynthesis/transport, phosphotransferase system (PTS), and the two‐component regulatory system between the four isolated NS strains and five strains that are phylogenetically close to the isolated GS strains from ground‐control soils. (F) Growth curves of four bacterial strains in liquid culture media with different concentrations of NaClO_4_. The OD_600_ values of pure liquid culture media were measured as the background for the values of growth curves.

Living bacterial strains were then isolated from near‐space‐exposed and nonexposed soil samples. For exposed desert soil samples, only one single colony was grown on R2A plates (strain NS4) but no colony was acquired on LB, TSA, and PCA media using Method I (see File S1: Materials and methods in the Supporting Information section), while >50 colonies (finally identified 10 bacterial populations named GS1–GS10) formed on various agar media (LB, R2A, and TSA) from nonexposed ground control samples using Method I (Table S2), indicating the severe decrease of living microbes in soils after near‐space exposure. To isolate more strains from near‐space‐exposed samples, postflight soils were precultured in liquid media using Method II (see File S1: Materials and methods in the Supporting Information section), and then additional three bacterial strains were successfully isolated from TSA plates (strains NS1, NS2, and NS3). The isolated strains formed pink (NS1), white (NS2 and NS4), and green (NS3) colonies on their respective agar plates after 3–7 days of incubation (Figure S2). All strains were rod‐shaped bacteria, with NS3 and NS4 (1–3 μm in length) being smaller than NS1 and NS2 (4–5 μm in length).

According to the phylogenetic analysis of 16S rRNA gene sequences, NS1 and NS2 were affiliated with the genera *Peribacillus* and *Bacillus*, respectively, in the phylum *Bacillota*, and NS3 and NS4 were affiliated with the genera *Cellulomonas* and *Pseudarthrobacter*, respectively, in the phylum *Actinomycetota* (Figure 1C). The BLAST analysis of 16S rRNA gene sequences demonstrated that strains NS1, NS2, NS3, and NS4 shared high similarities (>99%) with the corresponding sequences of *Peribacillus frigoritolerans* (NS1), *Bacillus atrophaeus* (NS2), *Cellulomonas cellasea* (NS3), and *Pseudarthrobacter enclensis* (NS4), respectively (Table S2). Strains isolated from nonexposed ground control soils were affiliated with the genera *Arthrobacter* (GS1–GS6 and GS9), *Nocardioides* (GS7), and *Pseudarthrobacter* (GS10) in the phylum *Actinomycetota* and the genus *Microvirga* (GS8) in the phylum *Pseudomonadota*, according to the analysis of 16S rRNA gene sequences (Table S2). The complete genomes of NS1, NS2, NS3, and NS4 were further sequenced and analyzed (Figure S3 and Table S3). The Average Nucleotide Identity and Average Amino Acid Identity (ANI/AAI) values between NS1 and its closest relative, *P. frigoritolerans* strain FJAT‐2396^T^, NS2 and *B. atrophaeus* strain NRRL NRS 213^T^, NS3 and *C. cellasea* strain NBRC 3753^T^, and NS4 and *Pseudarthrobacter phenanthrenivorans* Sphe3^T^ were 96.83%/96.99%, 98.46%/98.23%, 90.89%/91.05%, and 83.25%/84.74%, respectively. Therefore, here, these four novel isolated strains were named *P. frigoritolerans* strain NS1, *B. atrophaeus* strain NS2, *Cellulomonas* sp. strain NS3, and *Pseudarthrobacter* sp. strain NS4 (Figure 1C,D).

The predicted metabolic models of four isolated NS strains were constructed according to the genomic analysis (Figure S4). In general, all four genomes contain almost all genes for glycolysis, gluconeogenesis, both oxidative and nonoxidative pentose phosphate pathways, and the citric acid cycle (TCA cycle). Various ABC transporter‐encoding genes involved in sugar transport are detected in all genomes, including those for glucose, mannose, ribose, α‐glucoside, maltose, and maltodextrin. All four genomes possess nitrite reductase‐encoding genes (*nirBD*) that catalyze the reduction of nitrite to ammonia, and chlorite dismutase‐encoding genes (*cld*) that catalyze the conversion of chlorite into chloride and oxygen. Only the NS1 genome encodes the aerobic carbon monoxide dehydrogenase (CoxSML) for carbon monoxide oxidation. Moreover, the NS2 and NS3 genomes harbor genes encoding perchlorate reductase subunits (PcrAB) that catalyze the reduction of perchlorate to chlorite, and genes for nitrate reductases (NarGH) that catalyze the reduction of nitrate to nitrite.

To better understand the potential survival strategies of the four NS strains isolated from exposed soils, stress response genes and pathways that may be involved in the response to extreme environmental conditions were analyzed in genomes. Additionally, we compared their functional characteristics with five strains that are phylogenetically close to GS strains isolated from ground control soils (Figures 1E and S5). Genomic analysis of four isolated NS strains revealed a group of stress response genes, including *uvrABC*, *mutSL*, *recA*, *des*‐*desKR*, *sigB*, *csp*‐family, and *dps*, which could help strains cope with DNA damage, low pressure, temperature fluctuation, and starvation (Figure S4). Kyoto Encyclopedia of Genes and Genomes modules involved in the compatible solute biosynthesis/transport, phosphotransferase systems (PTSs), and the two‐component regulatory systems were further investigated (Figure 1E). The biosynthesis and transport of compatible solutes, including betaine and trehalose, are important salt‐out strategies for cells to prevent osmotic stress^16^, which could enable the NS strains to tolerate near‐space desiccation and Mars‐like hypersaline conditions. In addition, the PTS found in the NS1 and NS2 strains is not only a carbohydrate uptake system but also plays a pivotal role in sensing nutrient fluctuation and other stressors^17^. A previous study found that long‐term spaceflight increased the biofilm formation ability and cell wall resistance to environmental stress of a *Staphylococcus warneri* strain, and further analysis suggested that these changes could be attributed to the upregulated gene expression of the PTS^18^. The two‐component regulatory systems found in the NS strains are key mediators of signal transduction and allow bacteria to respond to multiple environmental stimuli^19^. A group of two‐component regulatory systems involved in stress response were found in the NS1, NS2, and NS3 strains, including those associated with the response to membrane lipid fluidity regulation, starvation, osmotic stress, and oxidative stress, implying that they have higher resistance to harsh conditions than those ground control strains. It should be noted that the spore‐forming *Peribacillus* and *Bacillus* are well known to cope with extreme terrestrial and extraterrestrial environments by forming endospores^20^. Thus, the NS1 and NS2 strains may survive the near‐space environments in their dormant states. Collectively, the formation of endospores, the stress‐sensing and ‐regulatory systems, stress response proteins, and the biosynthesis and transport of compatible solutes may contribute to the survival of the NS strains under near‐space Mars‐like environments.

In addition to extreme environmental conditions, relatively high quantities of perchlorates have been identified on the surface of Mars, and these salts would greatly influence the habitability of present‐day Mars^11^, ^12^. In this study, the four isolated NS strains were shown to have compatible solute biosynthesis and transport pathways, two‐component regulatory systems involved in the response to osmotic stress, and perchlorate/chlorite reduction genes, indicating their potential resistance to high‐perchlorate conditions. Therefore, we experimentally assessed the perchlorate tolerance of the isolated NS strains to investigate whether they could adapt to high‐perchlorate conditions. Although increased perchlorate concentrations inhibit the growth rate of strains to some extent, all four NS strains were perchlorate‐tolerant and could grow at concentrations of up to 150 mM NaClO_4_ (Figure 1F). Among them, NS2 was the most perchlorate‐tolerant strain and could grow at 500 mM perchlorate after a relatively long lag phase. NS1 was resistant to perchlorate up to 300 mM, but the growth rate was inhibited. NS3 and NS4 had lower perchlorate tolerance than strains NS1 and NS2, but they could still survive up to a concentration of 150 mM NaClO_4_ (Figure 1F). These results demonstrated that the four isolated strains are tolerant to high perchlorate concentration and could be optimal candidates for further astrobiological research.

Overall, the four isolated bacterial strains from near‐space‐exposed soils not only show tolerance to Mars‐like harsh environmental conditions but also have a high tolerance to perchlorate solutions. It should be noted that the exposure time for soil samples to the near space in this study was only 4 h. Therefore, whether the isolated strains could tolerate longer terms of exposure remains to be further analyzed. Nevertheless, this study demonstrates the successful application of near space for screening extremophilic bacterial strains capable of withstanding short‐term polyextreme environments. The four newly isolated bacterial strains are promising candidates for future astrobiological research, such as exposure experiments on space stations or in laboratory‐based planetary environment simulation chambers.

## AUTHOR CONTRIBUTIONS

**Li Liu**: Conceptualization (equal); data curation (equal); formal analysis (lead); investigation (lead); methodology (equal); visualization (equal); writing—original draft (lead). **Mengling Kang**: Investigation (equal); writing—review and editing (equal). **Zhe Wang**: Investigation (equal); writing—review and editing (equal). **Jianxun Shen**: Writing—review and editing (equal). **Yongxin Pan**: Investigation (equal); writing—review and editing (equal). **Wei Lin**: Conceptualization (equal); data curation (equal); funding acquisition (lead); methodology (equal); project administration (lead); supervision (lead); validation (equal); writing—review and editing (lead).

## ETHICS STATEMENT

This study did not involve any human participants or animal subjects.

## CONFLICT OF INTERESTS

The authors declare no conflict of interests.

## Supporting information

Supporting information.

## Data Availability

The complete genomes have been deposited under the NCBI BioProject PRJNA869781 with accession numbers CP103288–103291. The 16S rRNA gene sequences have been deposited at NCBI under accession numbers OP247653–247656 and OP316902–316911.
