# Supplementary material for: Perchlorate‐tolerant bacterial strains isolated from the Mars‐analog Qaidam Basin soils exposed to Earth's near space
Source: mLife. 2024 Sep 16;3(3):471–5. doi: 10.1002/mlf2.12142 (PMC11442124; doi:10.1002/mlf2.12142)
Supplement: Supplementary file 1 — Supporting information. [file MLF2-3-471-s001.docx]

**Supporting** **Information**

**Perchlorate-tolerant bacterial strains isolated from the Mars-analog Qaidam Basin soils exposed to Earth’s near space**

Li Liu^1,2^, Mengling Kang^1,2^, Zhe Wang^1,2^, Jianxun Shen^1^, Yongxin Pan^1,2^, Wei Lin^1,2*^

^1^Key Laboratory of Earth and Planetary Physics, Institute of Geology and Geophysics, Chinese Academy of Sciences, Beijing 100029, China.

^2^College of Earth and Planetary Sciences, University of Chinese Academy of Sciences, Beijing 100049, China.

*E-mail address: weilin@mail.iggcas.ac.cn

Running title: Bacteria isolated from near-space exposed soils

**MATERIALS AND METHODS**

**Desert soil collection and exposure sample preparation**

The selected sampling site was located at Dachaidan within the Qaidam Basin, northern Tibetan Plateau, China (37.74°N, 95.34°E). Soil samples were collected one day before the launch of the flight mission. Regolith within 10 cm depth was collected using sterilized scoops and stored in 50-ml sterile centrifuge tubes. Then, about 6 g of soil was transferred into a non-airtight type sample container (61 mm in diameter and 20 mm in height) with a 0.22-μm sterile filter on the bottom, and the final thickness of the soil was approximately 6 mm. All sample containers were stored at 4℃ until being mounted onto the BIOSEP at night. Each sample container was covered with a UV-grade quartz glass (Tangsinuo, China) on the top that enabled >85% transmission at the wavelength of 200 nm and >91% at 210nm-280nm, and a 0.22-μm filter at the bottom that enabled the protection of samples from external contaminations and the connection of the internal environment of each container with the ambient near space atmosphere.

**Balloon payload and near space flight experiment**

Under the support of the Scientific Experimental System in Near Space (SENSE) project initiated by the Chinese Academy of Sciences (CAS), the CAS-BAP was designed to conduct astrobiological research in Earth’s lower near space (1). The flight mission HH-21-5 was performed at Dachaidan (37.74°N, 95.34°E) in Qinghai Province close to the sampling site on September 17, 2021 at a float altitude of 35 km for 4 h 11 min. Triplicate soil samples were exposed to multiple extreme conditions of coldness, desiccation, low pressure, and high levels of radiation in the platform float area, and ground controls in triplicate were kept for the same period at 4°C. The sample containers onboard the BIOSEP allowed biological samples to be exposed to near space radiation through the quartz windows as well as the ambient atmosphere through the 0.22-μm sterile filters. All sample containers were successfully recovered, stored at 4°C, and transported to the laboratory for further processing.

**Description of near space balloon flight**

A total of 30 sample containers were mounted to the sample mounting plate in the BIOSEP, which was mounted on the CAS-BAP at an elevation angle of 30° relative to the horizon. The BIOSEP was launched at 7:31 a.m. China Standard Time (CST) on September 17, 2021 and uplifted to a float altitude of 35 km using a scientific helium balloon (80,000 m^3^) with a total flight time of 7 h. The total float time at 35 km was 4 h 11 min. The shade cover of BIOSEP was opened at 9:49 a.m. and closed at 1:49 p.m. CST, with the exposure period lasting for 4 hours. Temperature data was measured by two sensors mounted on the CAS-BAP and the BIOSEP, respectively. During the exposure period, the surface temperatures of CAS-BAP ranged from -22.39°C to 11.14°C and the internal temperatures of sample containers ranged from -21.5°C to 49.1°C. It should be noted that the measured temperatures increased due to continuous exposure of the platform and payload surface to intense sunlight. As a result, the later data could not represent the true near space ambient temperature. UV radiation was not measured during this flight mission.

**Isolation and cultivation of microbes**

The surfaces of all sample containers were first carefully cleaned with 75% ethanol to avoid any contaminations before sample processing. Then, microbes in post-exposed soil samples and non-exposed ground controls were isolated and cultured after soil homogenization. The culture method Ⅰ was first used for both types of soils to isolate surviving strains and compare the numbers of survivors, while the modified method Ⅱ was subsequently used for post-exposed soils to isolate additional surviving microbes. (Ⅰ) About 0.5 g of soil sample was mixed with 20 ml PBS and vortexed at 3,000 rpm for 30 min. After centrifugation at 1,500 rpm for 15 min, the supernatant was serially diluted (from 10^-1^ to 10^-3^), and 200 μl of each supernatant was spread on agar media plates. To incubate soil microbes, eight types of agar media (15% agar) R2A, 0.2 × R2A, Tryptic Soy Agar (TSA), 0.2 × TSA, Luria-Bertani (LB), 0.2 × LB, Plate Count Agar (PCA), and 0.2 × PCA were prepared. Plates were incubated at 26°C in dark until colonies formed. Subsequently, all colonies were streaked on new plates and incubated at 26°C in dark until visible colonies formed. (Ⅱ) About 0.25 g of soil sample was mixed with 1.5 ml of Tryptic Soy Broth (TSB) medium and incubated at 26°C shaking at 200 rpm for 12 h. The supernatant was serially diluted (from 10^-1^ to 10^-4^), and 200 μl of supernatant was used to spread on TSA (15% agar) plates. Plates were incubated at 26°C in dark until colonies formed. Subsequently, all colonies were streaked on new TSA plates and incubated at 26°C in dark.

**Scanning electron microscopy (SEM) and transmission electron microscopy (TEM) analyses**

After visible colonies appeared on plates, these cells were resuspended and washed three times with double-deionized water (ddH_2_O) and subsequently deposited on silica wafers or copper grids for air drying. The morphology of cells was examined by SEM (Thermo Scientific Apreo SEM, Thermo Fisher Scientific, USA) at 5 kV and by TEM (JEM-2100HR TEM, JEOL Ltd., Japan) at 200 kV.

**16S rRNA gene amplification and sequencing**

Pure cultured cells were suspended in ddH_2_O in a 1.5-ml centrifuge tube. The tube was placed in boiling water for 5 min of cell lysis and the supernatant was used as the DNA template for polymerase chain reaction (PCR) operations. About 1.5-kb 16S ribosomal RNA (rRNA) gene sequences were amplified using the bacterial universal primers 27F (5’-AGAGTTTGATCCTGGCTCA-3’) and 1492R (5’-GGTTACCTTGTTACGACTT-3’) through an initial denaturation at 95°C for 5 min; followed by 30 cycles of 30 s at 95°C, 30 s at 55°C and 90 s at 72°C; the final extension was carried out at 72°C for 10 min. The 20-μl PCR system contained 1 μl DNA template, 10 μl DreamTaq Green PCR Master Mix (2 ×) (Thermo Scientific, Thermo Fisher, USA), 0.5 μl forward primer (10 μM), 0.5 μl reverse primer (10 μM), and 8 μl ddH_2_O. PCR products were sequenced by Sanger sequencing at Huada Gene Company (BGI, Beijing, China).

The merged 16S rRNA gene sequences were analyzed using BLASTN (https://blast.ncbi.nlm.nih.gov/Blast.cgi). Sequence alignment was performed using ClustalW in MEGA11 (2) and a maximum likelihood phylogenetic tree was constructed using IQ-TREE (version 1.6.9) (3) under the TN+F+R3 substitution model with 1,000 ultrafast bootstraps. The phylogenetic tree was rooted with *Thermotoga petrophila* RKU-1^T^ and *Thermotoga maritima* MSB8^T^ belonging to the *Thermotogae* phylum. The final tree was visualized and modified using the Interactive Tree of Life (iTOL) (4).

**Whole genome sequencing and analysis**

Genomic DNA was extracted using the sodium dodecyl sulfate (SDS) method as previously described (5). The whole genomic DNA was sequenced using a combination of PacBio and Illumina NovaSeq PE150 platforms (Beijing Novogene Bioinformatics Technology Co., Ltd). The PacBio reads were assembled using SMRT Link v5.0.1 and then polished by Arrow using the Illumina reads (6). The completeness and contamination of assembled genomes were evaluated using CheckM (7). To predict the gene functions of the whole genomes, Clusters of Orthologous Groups (COG), Kyoto Encyclopedia of Genes and Genomes (KEGG), and Gene Ontology (GO) databases were used to conduct BLAST search (E-value <1e-5, minimal alignment length percentage >40%). Coding sequences were predicted using GeneMarkS v4.17 (8). tRNAscan-SE v1.3.1 (9) and rRNAmmer v1.2 (10) were used to predict transfer RNA (tRNA) and rRNA, respectively, and the Rfam database (11) was used to predict small RNA (sRNA). Genomic information was visualized using the CGView server (12). Genomic taxonomic classification was conducted using the Genome Taxonomy Database Toolkit (GTDB-Tk version 0.3.2; database release R04-RS89) (13).

Phylogenetic analysis of microbial genomes was performed using PhyloPhlAn (version 3.0.58) (14). Reference genomes were selected from the GTDB (database release R04-RS89) (15) (GTDB species representatives only and NCBI type material only) and then downloaded from the NCBI database. “Phylophlan” database (400 optimized universal marker genes) and default configuration “supermatrix_aa.cfg” were used. The diversity parameter was set to “low”. The phylogenomic tree was rooted with *Thermotoga petrophila* RKU-1^T^ and *Thermotoga maritima* MSB8^T^ belonging to the *Thermotogae* phylum. The final tree was visualized and modified using iTOL (4). Average nucleotide identity (ANI) and average amino acid identity (AAI) values were calculated using enveomics (16).

The metabolic capabilities of whole genomes were classified using METABOLIC v4.0 (17). The “KEGGModuleHit” results were used to identify the functional characteristics of genomes. Five strains that are phylogenetically close to isolated strains from ground-control soils were included in the analysis of the functional characteristics, including *Nocardioides psychrotolerans* NBRC 108563 (GenBank accession BKAF01000001.1), *Pseudarthrobacter siccitolerans* strain 4J27 (GenBank accession CAQI01000001.1), *Microvirga aerilata* strain 5420S (GenBank accession JAEQMY010000100.1), *Arthrobacter tumbae* strain DSM 16406 (GenBank accession JAFBCC010000001.1), and *Arthrobacter ruber* strain MDB1.42 (GenBank accession PPTH01000001.1). The metabolic pathways found in one or both of the “FunctionHit” and “KEGGModuleHit” results were used to reconstruct cell metabolic models. Putative proteins related to stress responses were further analyzed using the RAST annotation server (18) with a cutoff E-value <1e-5.

**Growth of isolated strains in different concentrations of perchlorate**

All four bacterial strains isolated from near-space exposed soils were cultured in liquid culture media containing different concentrations of sodium perchlorate (NaClO_4_) (0, 20, 40, 80, 150, 300, and 500 mM). Twenty-four well cell culture plates were used, with each well containing 1 ml liquid culture media, in triplicate for each culture system. Blank liquid culture media were used as negative controls. All 24-well plates were sealed with parafilm after inoculation and then incubated in dark at 26°C, shaking at 54 rpm. Growth curves of cells (OD_600_) were measured using a SpectraMax i3 (Molecular Devices, CA, USA). The OD_600_ values of blank liquid culture media were measured as background for the values of growth curves.

**Table S1.** Environmental conditions of near space (~ 35 km ASL) compared to Martian surface ^a^.

|  | Near space | Martian surface |
| --- | --- | --- |
| Average temperature | ~-36°C | ~-67°C |
| UV radiation | ~100 W/m^2^ | ~50 W/m^2^ |
| Ionizing radiation | ~0.1 mGy/d | ~0.2 mGy/d |
| Atmospheric pressure | <1 kPa | ~ 0.7 kPa |
| Relative humanity | <1% | / |

^a^ Data was collected from previous studies (19-21).

**Table S2.** Taxonomic classifications of the four NS strains and ten bacterial strains isolated from non-exposed ground control soils according to BLASTN analysis of 16S rRNA gene sequences.

| Strain | Medium | Best BLASTN hit | Phylum | Identity (%) |
| --- | --- | --- | --- | --- |
| NS1 | TSA | *Peribacillus frigoritolerans*  (MT065803.1) | *Bacillota* | 100.00 |
| NS2 | TSA | *Bacillus atrophaeus*  (MN826517.1) | *Bacillota* | 100.00 |
| NS3 | TSA | *Cellulomonas cellasea*  (JF496362.1) | *Actinomycetota* | 99.93 |
| NS4 | R2A | *Pseudarthrobacter enclensis*  (OL824810.1) | *Actinomycetota* | 99.71 |
| GS1 | LB | *Arthrobacter ruber*  (OP080779.1) | *Actinomycetota* | 99.86 |
| GS2 | LB | *Arthrobacter tumbae*  (MK696393.1) | *Actinomycetota* | 99.93 |
| GS3 | LB | *Arthrobacter tumbae*  (OK298990.1) | *Actinomycetota* | 99.78 |
| GS4 | LB | *Arthrobacter tumbae*  (MK696393.1) | *Actinomycetota* | 99.93 |
| GS5 | R2A | *Arthrobacter ruber*  (OQ255435.1) | *Actinomycetota* | 99.93 |
| GS6 | R2A | *Arthrobacter ruber*  (OQ255435.1) | *Actinomycetota* | 99.93 |
| GS7 | R2A | *Nocardioides psychrotolerans*  (NR_109408.1) | *Actinomycetota* | 97.86 |
| GS8 | R2A | *Microvirga aerilata*  (NR_114298.1) | *Pseudomonadota* | 98.89 |
| GS9 | TSA | *Arthrobacter tumbae*  (MK696393.1) | *Actinomycetota* | 99.93 |
| GS10 | TSA | *Pseudarthrobacter siccitolerans*  (MF681872.1) | *Actinomycetota* | 99.78 |

**Table S3.** General genomic features of four isolated bacteria strains of *Peribacillus frigoritolerans* strain NS1 (NS1), *Bacillus atrophaeus* strain NS2 (NS2), *Cellulomonas* sp. strain NS3 (NS3), and *Pseudarthrobacter* sp. strain NS4 (NS4).

|  | NS1 | NS2 | NS3 | NS4 |
| --- | --- | --- | --- | --- |
| Culture medium | TSA | TSA | TSA | R2A |
| Genome size (Mb) | 5.54 | 4.26 | 4.78 | 4.44 |
| Number of contigs | 1 | 1 | 1 | 1 |
| Completeness (%) | 98.91 | 99.17 | 99.42 | 99.71 |
| Contamination (%) | 1.39 | 0 | 0 | 0.15 |
| GC content (%) | 40.61 | 43.33 | 74.68 | 64.55 |
| Number of coding sequences | 5,914 | 4,414 | 4,384 | 4,254 |
| Number of tRNAs | 84 | 83 | 45 | 50 |
| Number of rRNAs | 42 | 24 | 6 | 15 |
| Number of sRNAs | 3 | 9 | 0 | 0 |
| Number of CRISPRs | 3 | 1 | 1 | 0 |
| Number of genes annotated  against COG | 3,891 | 3,171 | 3,103 | 3,070 |
| Number of genes annotated  against KEGG | 4,946 | 4,066 | 3,826 | 3,881 |
| Number of genes annotated  against GO | 3,673 | 2,831 | 2,688 | 2,682 |


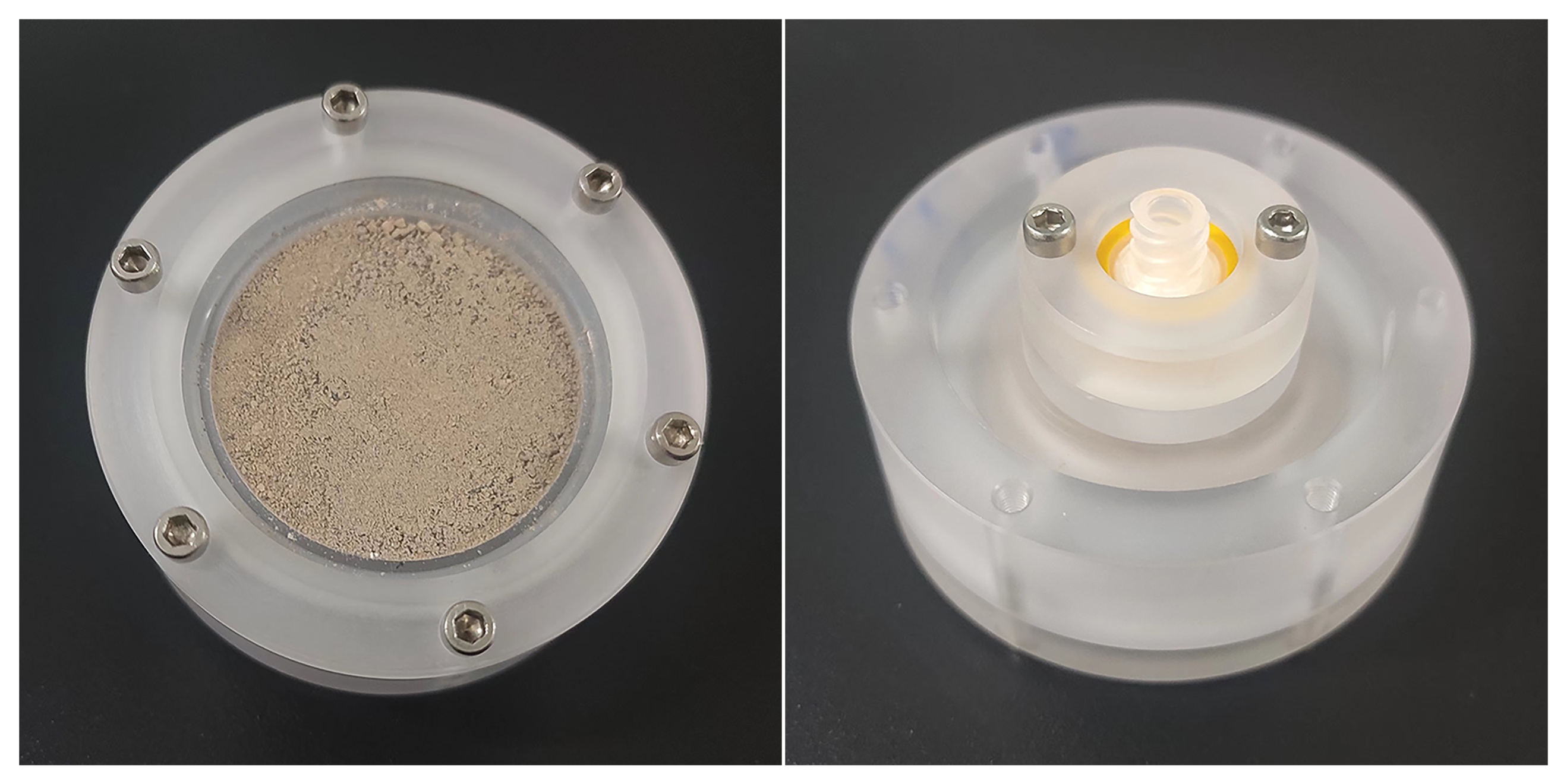


**Figure S1.** Photographs of the sample container. Each sample container was covered with a UV-grade quartz glass on the top that enabled >85% transmission at the wavelength of 200 nm and >91% at 210nm-280nm (left). A 0.22-μm filter was mounted at the bottom that enabled the protection of samples from outer contamination and the connection of the internal space of each container with the ambient atmosphere (right).


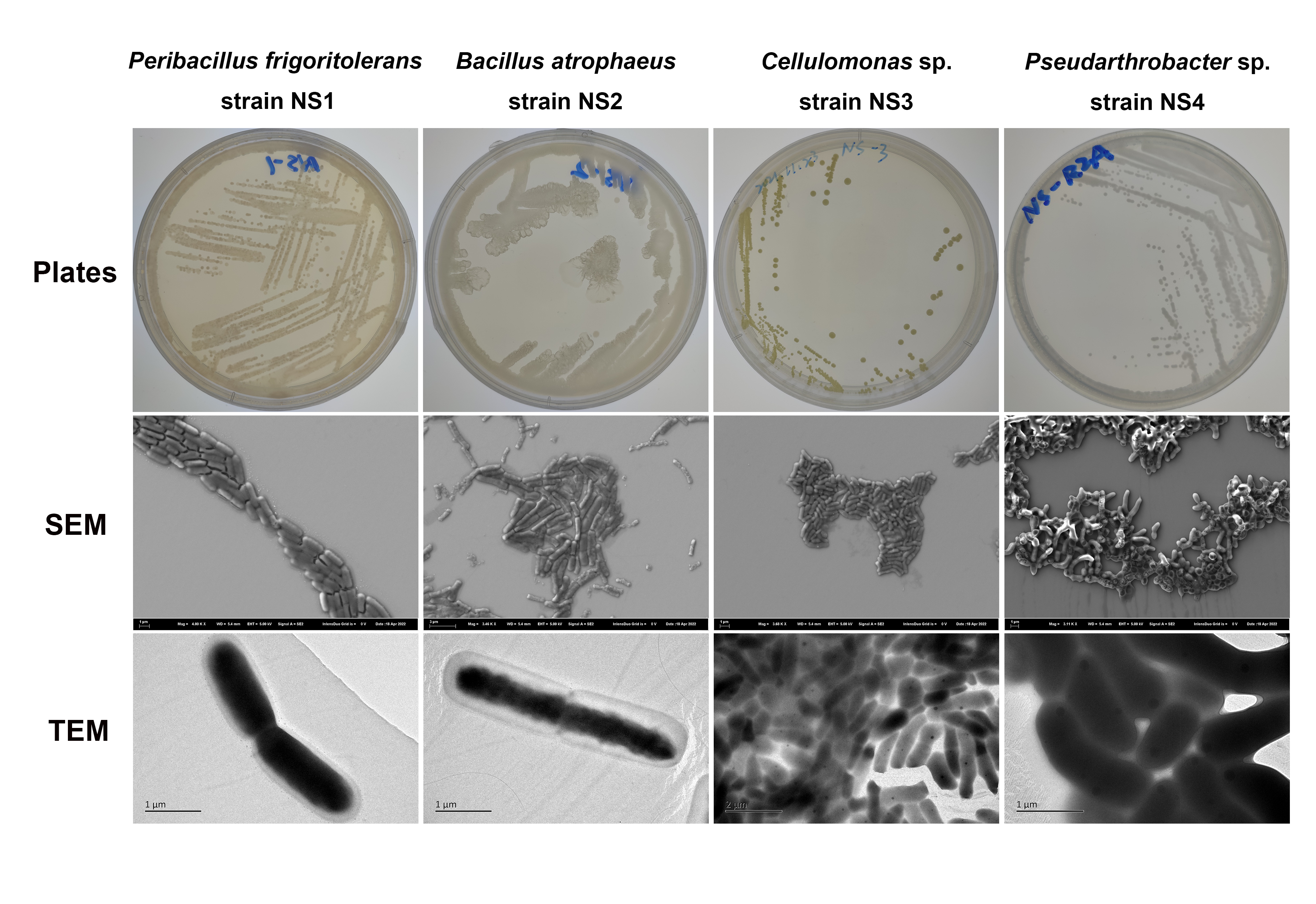


**Figure S2.** Images of isolated pure culture colonies on TSA (*Peribacillus frigoritolerans* strain NS1, *Bacillus atrophaeus* strain NS2, and *Cellulomonas* sp. strain NS3) and R2A (*Pseudarthrobacter* sp. strain NS4) agar plates, and scanning electron microscopy (SEM) and transmission electron microscopy (TEM) images for NS1, NS2, NS3, and NS4 cells.


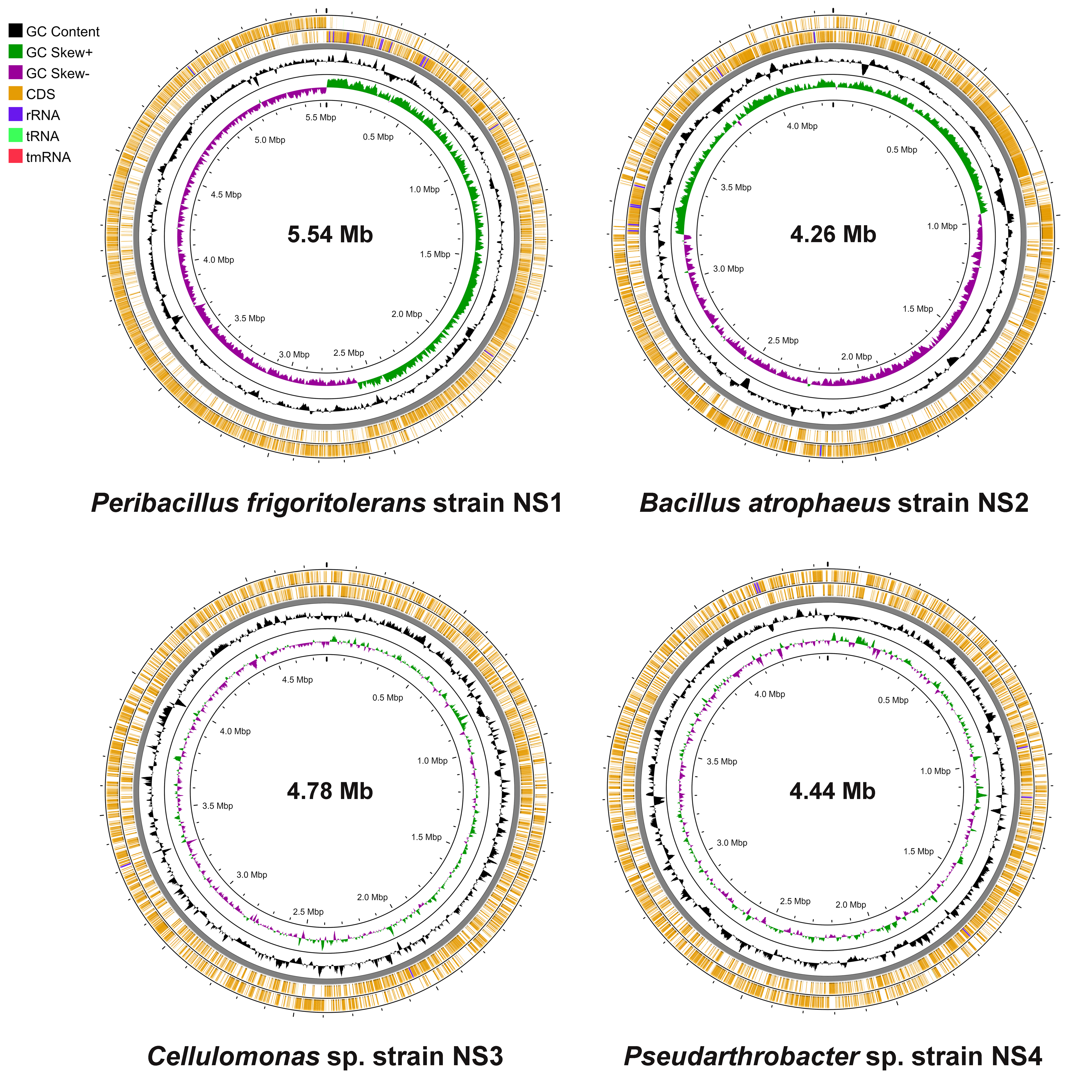


**Figure S3.** Circular whole-genome maps of four isolated bacteria NS1, NS2, NS3, and NS4. The genomes were reconstructed using the PacBio and Illumina NovaSeq PE150 platforms. Detailed information of genomes from the innermost circle to the outermost were (1) the size of the genome, (2) GC skew (purple: G%<C%; green: G%>C%), (3) GC content (inward: <average GC content; outward: >average GC content) and (4) coding sequences (CDS) on the forward and reverse strands, respectively.


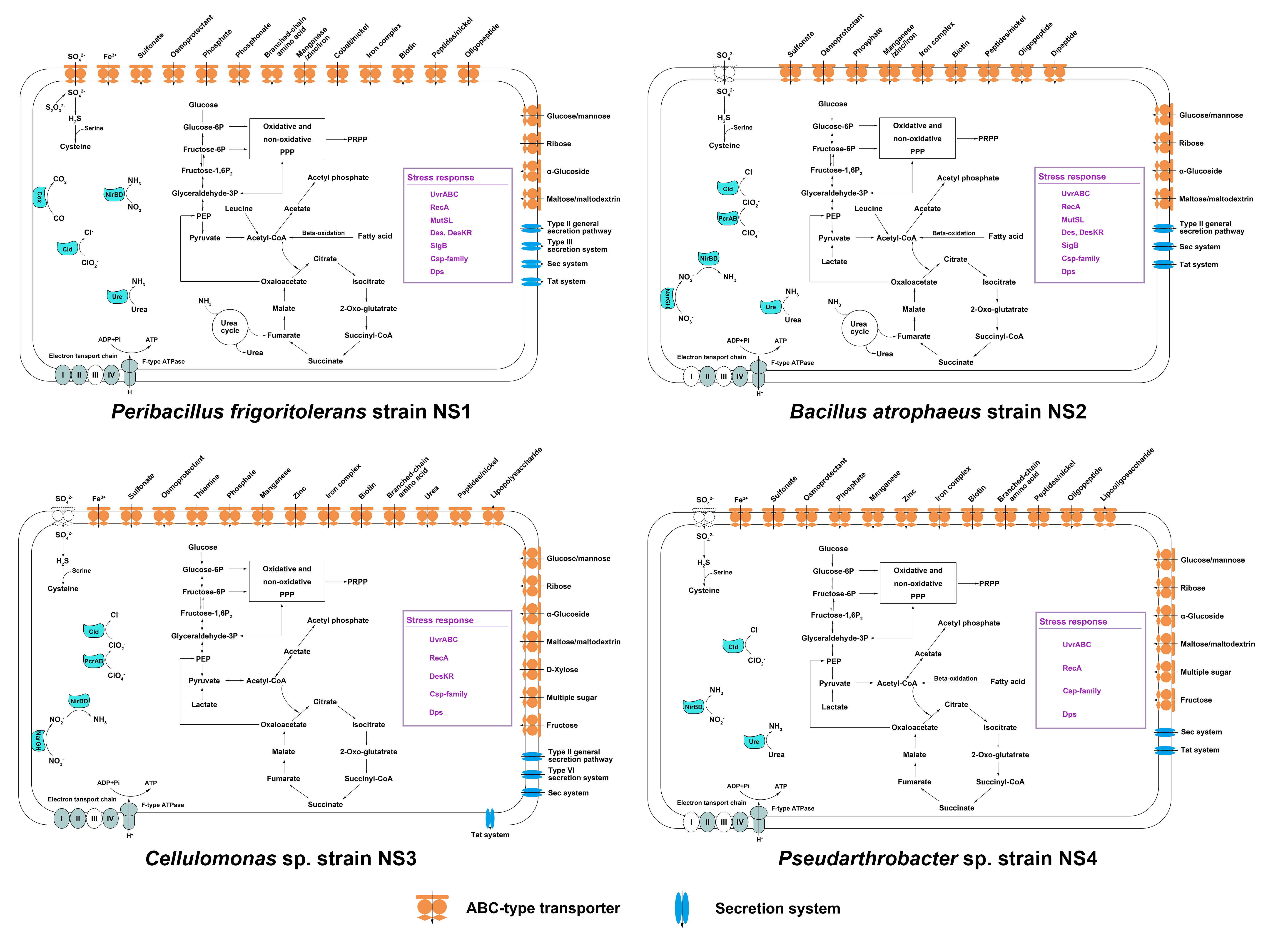


**Figure S4.** Predicted metabolic models of strains NS1, NS2, NS3, and NS4. Dash lines or grey arrows represent the absence of the enzyme or the pathway. UvrABC, excinuclease ABC subunits ABC; RecA, DNA recombination/repair protein; MutSL, DNA mismatch repair protein; Des, membrane fatty acid desaturase; DesK, sensor histidine kinase; DesR, transcriptional response regulator; SigB, RNA polymerase sigma factor; Csp-family, cold shock protein family; Dps, DNA starvation/stationary phase protection protein.

**Figure S5.** Functional comparison between near-space exposed strains and five strains that are phylogenetically close to isolated GS strains from ground-control soils. A total of 304 KEGG modules out of M00001-M00819 were found in the genomes.

**REFERENCES**

1. Lin W, He F, Zhang W, Yao Z, Shen J, Ren Z, et al. Astrobiology at altitude in Earth's near space. Nat Astron. 2022;6(2):289.

2. Tamura K, Stecher G, Kumar S. MEGA11: Molecular Evolutionary Genetics Analysis Version 11. Mol Biol Evol. 2021;38(7):3022-7.

3. Nguyen L-T, Schmidt HA, von Haeseler A, Bui Quang M. IQ-TREE: A fast and effective stochastic algorithm for estimating maximum-likelihood phylogenies. Mol Biol Evol. 2015;32(1):268-74.

4. Letunic I, Bork P. Interactive Tree Of Life (iTOL) v4: recent updates and new developments. Nucleic Acids Res. 2019;47(W1):W256-W9.

5. Lim HJ, Lee EH, Yoon Y, Chua B, Son A. Portable lysis apparatus for rapid single-step DNA extraction of Bacillus subtilis. J Appl Microbiol. 2016;120(2):379-87.

6. Ardui S, Ameur A, Vermeesch JR, Hestand MS. Single molecule real-time (SMRT) sequencing comes of age: applications and utilities for medical diagnostics. Nucleic Acids Res. 2018;46(5):2159-68.

7. Parks DH, Imelfort M, Skennerton CT, Hugenholtz P, Tyson GW. CheckM: assessing the quality of microbial genomes recovered from isolates, single cells, and metagenomes. Genome Res. 2015;25(7):1043-55.

8. Besemer J, Lomsadze A, Borodovsky M. GeneMarkS: a self-training method for prediction of gene starts in microbial genomes. Implications for finding sequence motifs in regulatory regions. Nucleic Acids Res. 2001;29(12):2607-18.

9. Lowe TM, Eddy SR. tRNAscan-SE: A program for improved detection of transfer RNA genes in genomic sequence. Nucleic Acids Res. 1997;25(5):955-64.

10. Lagesen K, Hallin P, Rodland EA, Staerfeldt H-H, Rognes T, Ussery DW. RNAmmer: consistent and rapid annotation of ribosomal RNA genes. Nucleic Acids Res. 2007;35(9):3100-8.

11. Gardner PP, Daub J, Tate JG, Nawrocki EP, Kolbe DL, Lindgreen S, et al. Rfam: updates to the RNA families database. Nucleic Acids Res. 2009;37:D136-D40.

12. Grant JR, Stothard P. The CGView Server: a comparative genomics tool for circular genomes. Nucleic Acids Res. 2008;36(Web Server issue):W181-4.

13. Chaumeil P-A, Mussig AJ, Hugenholtz P, Parks DH. GTDB-Tk: a toolkit to classify genomes with the Genome Taxonomy Database. Bioinformatics. 2020;36(6):1925-7.

14. Asnicar F, Thomas AM, Beghini F, Mengoni C, Manara S, Manghi P, et al. Precise phylogenetic analysis of microbial isolates and genomes from metagenomes using PhyloPhlAn 3.0. Nat Commun. 2020;11(1):2500.

15. Parks DH, Chuvochina M, Chaumeil P-A, Rinke C, Mussig AJ, Hugenholtz P. A complete domain-to-species taxonomy for Bacteria and Archaea. Nat Biotechnol. 2020;38(9):1079-86.

16. Rodriguez-R LM, Konstantinidis KT. The enveomics collection: a toolbox for specialized analyses of microbial genomes and metagenomes. PeerJ Prepr. 2016;4:e1900v1.

17. Zhou Z, Tran PQ, Breister AM, Liu Y, Kieft K, Cowley ES, et al. METABOLIC: high-throughput profiling of microbial genomes for functional traits, metabolism, biogeochemistry, and community-scale functional networks. Microbiome. 2022;10(1):33.

18. Aziz RK, Bartels D, Best AA, DeJongh M, Disz T, Edwards RA, et al. The RAST server: Rapid annotations using subsystems technology. BMC Genomics. 2008;9:75.

19. Cortesão M, Siems K, Koch S, Beblo-Vranesevic K, Rabbow E, Berger T, et al. MARSBOx: Fungal and bacterial endurance from a balloon-flown analog mission in the stratosphere. Front Microbiol. 2021;12:601713.

20. Schuerger AC, Mancinelli RL, Kern RG, Rothschild LJ, McKay CP. Survival of endospores of Bacillus subtilis on spacecraft surfaces under simulated martian environments: implications for the forward contamination of Mars. Icarus. 2003;165(2):253-76.

21. Smith DJ, Sowa MB. Ballooning for biologists: Mission essentials for flying life science experiments to near space on NASA large scientific balloons. Gravitational Sp Res. 2017;5(1):52-73.
